# Thanks to all those who reviewed for *Trials* in 2015

**DOI:** 10.1186/s13063-016-1209-4

**Published:** 2016-02-15

**Authors:** Douglas G. Altman, Curt D. Furberg, Jeremy M. Grimshaw

**Affiliations:** Centre for Statistics in Medicine, University of Oxford, Botnar Research Centre, Windmill Road, Oxford, OX3 7LD UK; Division of Public Health Sciences, Wake Forest University School of Medicine, Medical Center Boulevard, Winston-Salem, 27157-1063 NC USA; Clinical Epidemiology Programme, Ottawa Hospital Research Institute and Department of Medicine, University of Ottawa, 501 Smyth Road, Ottawa, K1H 8 L6 ON Canada

## Abstract

A peer-reviewed journal would not survive without the generous time and insightful comments of the reviewers, whose efforts often go unrecognized. Although final decisions are always editorial, they are greatly facilitated by the deeper technical knowledge, scientific insights, understanding of social consequences, and passion that reviewers bring to our deliberations. For these reasons, the Editors-in-Chief and staff of *Trials* warmly thank the 854 reviewers whose comments helped to shape the journal, for their invaluable assistance with review of manuscripts in Volume 16 (2015).

**Isabel Adeyemi**

UK

**Rakesh Aggarwal**

India

**Vineet Ahuja**

India

**Bafeta Aïda**

France

**Rubens Albuquerque**

Brazil

**Rawan AlHeresh**

USA

**Kelli Allen**

USA

**Peter Allmark**

UK

**Terje Alraek**

Norway

**Cinthia Amorim**

Brazil

**Karin Amrein**

Austria

**Guipeng An**

China

**Martin Anders**

Czech Republic

**Susan Andersen**

Denmark

**Joachim Andrassy**

Germany

**Sharon Andrew**

UK

**Gavin Andrews**

Bahamas

**Ane Appelt**

Denmark

**Yvan Arlettaz**

Switzerland

**Nigel Armfield**

Australia

**Joanna Armstrong Schellenberg**

UK

**Jeffrey K Aronson**

UK

**Bérengère Aubry-Rozier**

Switzerland

**Susan Aucott**

USA

**Karen Austrian**

Kenya

**Cristina Avendaño**

Spain

**Nassim Ayoub**

Germany

**Samy Azer**

Saudi Arabia

**Simon Bacon**

Canada

**George Bagias**

Greece

**Ivan Bajsic**

Slovenia

**Liz Baker**

UK

**Christine Baldwin**

UK

**Karla Ballman**

USA

**Joyce Balls-Berry**

USA

**Carlos Bandeira de Mello**

Brazil

**Saber Davide Barbar**

France

**Alexandre Barbosa**

Brazil

**Karen Barker**

UK

**Pilar Barnestein-Fonseca**

Spain

**Rubina Barolia**

Pakistan

**Partha Basu**

India

**Erika Baum**

Germany

**Annette Becker**

Germany

**Yves Becouarn**

France

**David Bekelman**

USA

**Robert Bell**

UK

**M Fernanda Bellolio**

USA

**Eduardo Bergel**

Argentina

**Vance Berger**

USA

**Donna Berry**

USA

**James Betts**

UK

**Zhaoxiang Bian**

Hong Kong

**Nina Biehal**

UK

**Nerea Bielsa**

Spain

**Egbert Biesheuvel**

Netherlands

**Eveline Bijlard**

Netherlands

**Laurent Billot**

Australia

**Ariella Binik**

UK

**Bojan Biocina**

Croatia

**Bernadette Biondi**

Italy

**Gill Black**

South Africa

**Martin Blakemore**

UK

**Amy Blakemore**

UK

**Jane Blazeby**

UK

**Charlotte Blease**

Ireland

**Christian Blickem**

UK

**Jonathan Boote**

UK

**Bijan Borah**

USA

**Mark Borthwick**

UK

**Emmanuel Boselli**

France

**Catherine Bouman**

Netherlands

**Isabelle Boutron**

France

**Peter Bower**

UK

**Julia Boyle**

UK

**Tonje Braaten**

Norway

**Patrick Brady**

USA

**Marco Braggion**

Italy

**Mantaj Brar**

Canada

**Hugues Brat**

Switzerland

**Sanjin Braut**

Croatia

**Aline Brennan**

Ireland

**Graham Brennan**

UK

**Daniel Bressington**

Hong Kong

**Ivan Bristow**

UK

**Laurent Brochard**

Canada

**Anna Brokmeier**

Germany

**Marie Brossar-Racine**

USA

**Deborah Brown**

UK

**Sarah Brown**

UK

**Iris Brunner**

Norway

**Deborah Buck**

UK

**Dario Bugada**

Italy

**Lorraine Buis**

USA

**Mark Bullimore**

USA

**Claudia Buntrock**

Netherlands

**Matthew Burg**

USA

**M Cairns**

UK

**Robin Callister**

Australia

**Michael Campbell**

UK

**Marion Campbell**

UK

**Joe Canner**

USA

**Joseph Canner**

USA

**Toby Capstick**

UK

**Xavier Carcopino**

France

**Valentina Cardi**

UK

**Dawn Carnes**

UK

**Christopher Carroll**

UK

**Martin Cartwright**

UK

**Laura Castleman**

USA

**Daiane Cerutti-Kopplin**

Brazil

**Xiaorong Chang**

China

**Robin Chatters**

UK

**Pei Chen**

China

**Dacan Chen**

China

**Yuanfang Chen**

China

**Jianping Chen**

Hong Kong

**Bin Cheng**

China

**Annabel Chen-Tournoux**

Canada

**Stephen Chester**

Australia

**Alessandro Chiarotto**

Netherlands

**Dixon Chibanda**

Zimbabwe

**Wai Tong Chien**

Hong Kong

**William CS Cho**

Hong Kong

**Sun-mi Choi**

Korea, South

**Jesper Frank Christensen**

Denmark

**Robin Christensen**

Denmark

**Antonio Cianci**

Italy

**David Clarke**

UK

**Jan Clarkson**

UK

**Brian Clausen**

Denmark

**John Cleland**

UK

**Elisabeth Coart**

Belgium

**Lizzie Coates**

UK

**Erik Cobo**

Spain

**Nicole Cockayne**

Australia

**Christopher Coffey**

USA

**Judith Cohen**

UK

**Dave Collett**

UK

**Clare Collins**

Australia

**Emilie Combet**

UK

**Christian Compagnone**

Italy

**Felix Compen**

Netherlands

**Lynda Constable**

UK

**Cindy Cooper**

UK

**Philip Cooper**

UK

**Simon Coulton**

UK

**Nancy Covell**

USA

**Peter Alan Coventry**

UK

**Helen Cox**

South Africa

**Susan Cox**

Canada

**Peter Craig**

UK

**Angela Craigie**

UK

**Joanna Crocker**

UK

**Christine Cronin**

USA

**Jennifer Cunningham-Erves**

USA

**Martha Curley**

USA

**Geoffrey Curran**

USA

**Elizabeth Curtis**

UK

**Alberto Dal Molin**

Italy

**Amanda Daley**

UK

**Philippa Dall**

UK

**Rafael Dal-Ré**

Spain

**Lynae Darbes**

USA

**Elizabeth Dartnall**

South Africa

**Eric Daugas**

France

**Gail Davey**

UK

**Tracey Davidson**

UK

**Geertruida H de Bock**

Netherlands

**Alan de Brauw**

USA

**Rocco de Filippis**

Italy

**Renate de Jongh**

Netherlands

**Jorge de la Torre**

USA

**Raphael de Souza**

Brazil

**Bradley de Vries**

Australia

**Chris Deery**

UK

**Daniela DeFrino**

USA

**Brendan Delaney**

UK

**Giorgio Della Rocca**

Italy

**Jacques Demotes**

France

**Martin Dennis**

UK

**Martin Denvir**

UK

**Sonja Derman**

Germany

**Timothy DeRouen**

USA

**Martin Descarreaux**

Canada

**Catherine D'Este**

Australia

**Jan D'Haese**

Germany

**Gianfranco Di Gennaro**

Italy

**Laura Diamond**

Australia

**Neal Dickert**

USA

**Markus K Diener**

Germany

**Benjamin Djulbegovic**

USA

**Peter Dodek**

Canada

**Gordon Doig**

Australia

**Feixia Dong**

China

**Tara Donker**

Australia

**Joseph Donnelly**

USA

**Harriet Downing**

UK

**Janine Dretzke**

UK

**Didier Dreyfuss**

France

**Lea Drye**

USA

**Joel Dubin**

Canada

**Lelia Duley**

UK

**Jo Dumville**

UK

**TA Duong Dinh**

Germany

**Deep Dutta**

India

**David Ellard**

UK

**Philipp Eller**

Austria

**Elham Emami**

Canada

**Stephan Emich**

Austria

**Sarah Engebretsen**

USA

**Steven Engebretson**

USA

**Mert Erkan**

Germany

**Bente Appel Esbensen**

Denmark

**Munira Essat**

UK

**Farhad Etezadi**

Iran

**Mohammed Ezzeldin**

Spain

**Giacomo Faldella**

Italy

**Paul Farrand**

UK

**Barbara Farrell**

UK

**James Feldman**

USA

**Natasha Fernandes**

Canada

**Frederick Ferris**

USA

**Shona Fielding**

UK

**Francis Finucane**

Ireland

**Christina Fitzner**

Germany

**Deborah Fitzsimmons**

Canada

**John Fletcher**

Australia

**Peter Fonagy**

UK

**Maelán Fontes Villalba**

Spain

**Christen Fornadel**

USA

**David Forster**

USA

**Laura Forsythe**

USA

**Charlie Foster**

UK

**Marlene Fransen**

Australia

**Rosanne Freak-Poli**

Australia

**Tobias Freund**

Germany

**Tim Friede**

Germany

**Lawrence Friedman**

USA

**Jordan Fulcher**

Australia

**Jens Gaab**

Switzerland

**Jakub Gajewski**

Ireland

**Timothy Ganey**

USA

**Silvio Garattini**

Italy

**Azucena Garcia Palacios**

Spain

**Hrvoje Gasparovic**

Croatia

**Sebastien Gibot**

France

**Lesley Gillespie**

New Zealand

**Fiona Gillison**

UK

**Michael Gionfriddo**

USA

**Timothy Girard**

USA

**Bruno Giraudeau**

France

**Anne-Marie Glenny**

UK

**Tove Godskesen**

Sweden

**Amit Goel**

India

**Henriette Golcher**

Germany

**Michael Goldfarb**

Canada

**Charlie Goldsmith**

Canada

**Tim Gomersall**

UK

**Javier Gonzalez**

UK

**José Antonio González Alastrué**

Spain

**Steve Goodacre**

UK

**Steven Goodman**

USA

**Philip Goodney**

USA

**Rebecca Gossage-Worrall**

UK

**Yann Goueffic**

France

**Norbert Graf**

Germany

**Nadine Graham**

Canada

**Aileen Grant**

UK

**Sean Grant**

USA

**Jocelyn Gravel**

Canada

**Celia Gregson**

UK

**Andrew Grieve**

UK

**James Griffin**

UK

**Xavier Griffin**

UK

**Francisco Gude**

Spain

**Hilary Gunn**

UK

**Kurinchi Gurusamy**

UK

**LB Haddad**

Brazil

**Terry Haines**

Australia

**Nigel Hall**

UK

**Deborah Hall**

UK

**Katherine Hall**

USA

**Daniel Hall**

USA

**Christopher Halloran**

UK

**Lisa Hampson**

UK

**Helen Handoll**

UK

**Catherine Hankey**

UK

**Nicola Harman**

UK

**Frank Harrell**

USA

**Kathleen Harrington**

USA

**Jan Hartvigsen**

Denmark

**Anna Hawkes**

Australia

**Jean Hay-Smith**

New Zealand

**Liyun He**

China

**L Heckel**

Australia

**Siew Wan Hee**

UK

**Anna Hegedus**

Switzerland

**Charles Heilig**

USA

**Karla Hemming**

UK

**Ronald Henry**

Netherlands

**Hans-Werner Hense**

Germany

**Moonseong Heo**

USA

**Peter Herbison**

New Zealand

**Sarah Hetrick**

Australia

**Spencer Hey**

Canada

**Grainne Hickey**

Ireland

**Leanne Hides**

Australia

**Paul Hindmarch**

UK

**Allison Hirst**

UK

**Andrew Hoel**

USA

**Achim Hoerauf**

Germany

**John Hoey**

Canada

**Michael Höfler**

Germany

**Justus Hofmeyr**

South Africa

**Dan Eik Høfsten**

Denmark

**Rolf Holle**

Germany

**Ian Holloway**

UK

**Soren Holm**

UK

**Liu Hongwei**

China

**Richard Hooper**

UK

**Dell Horey**

Australia

**Kim Houlind**

Denmark

**Michael Howley**

USA

**Yen-Hsuan Hsu**

Taiwan

**Xi-Chun Hu**

China

**Gill Hubbard**

UK

**Adwoa Hughes-Morley**

UK

**John Hustad**

USA

**Melinda Hutchesson**

Australia

**Cathy Hutchison**

UK

**Wataru Ichikawa**

Japan

**Olubukola Idoko**

Gambia

**Joanne Ingwall**

USA

**Talia Isaacs**

UK

**Shariful Islam**

Australia

**Khalida Ismail**

UK

**Saki Ito**

USA

**Patricia Jabre**

France

**Rod Jackson**

New Zealand

**Insoo Jang**

Korea, South

**Mark Jayes**

UK

**Micoulaud Franchi Jean-Arthur**

France

**Gerald Jerome**

USA

**Guang Ji**

Ghana

**Fei Jiang**

USA

**Yu Jiang**

USA

**Bernd Jilma**

Austria

**Kent Johnson**

Australia

**Derek Johnson**

USA

**Kate Jolly**

UK

**Patricia Jones**

USA

**Maris Jones**

USA

**Sue Jordan**

UK

**Harald Thune Jorstad**

Netherlands

**Samir Joshi**

India

**Rohina Joshi**

Australia

**Andrew Judge**

UK

**Jon Jureidini**

Australia

**Suneetha Kadiyala**

UK

**Yega Kalairajah**

UK

**Thomas Kaley**

USA

**Lisa Kammerman**

USA

**Tammy Kammin**

USA

**Mona Kanaan**

UK

**Jothy Kandasamy**

UK

**Didem Karadibak**

Turkey

**Steve Karas**

USA

**Igor Karp**

Canada

**Goulnar Kasymjanova**

Canada

**Artur Katz**

Brazil

**Jay Katz**

USA

**Thomas Kaulhausen**

Germany

**Carol Keane**

Australia

**Richard Keefe**

USA

**Patrick Kelley**

USA

**Deanna Kelly**

USA

**Joanne Kemp**

Australia

**Stacey Kenfield**

USA

**Anne Kennedy**

UK

**Cassandra Kenning**

UK

**Samantha Keogh**

Australia

**Sally Kerry**

UK

**Martin Keszler**

USA

**Jyoti Khadka**

Australia

**Yoon Bum Kim**

Korea, South

**Hee-Soo Kim**

Korea, South

**Tae-Hun Kim**

Korea, South

**David Kimhy**

USA

**Bo Juel Kjeldsen**

Denmark

**Annet Kleiboer**

Netherlands

**Tobias Kleinjung**

Switzerland

**Shahnaz Klouche**

France

**Phillip Knebel**

Germany

**Emily Knight**

Canada

**Kristian Kniha**

Germany

**Robin Kok**

Netherlands

**Esther Kok**

UK

**Bo Kong**

Germany

**Andre Konski**

USA

**Daniel Kotz**

Netherlands

**Gabriel Krastl**

Germany

**Tobias Krieger**

Switzerland

**Anton Krige**

UK

**Lewis Kuller**

USA

**Merethe Kumle**

Norway

**Seungwon Kwon**

Korea, South

**Christopher Labos**

Canada

**Eduardo Lagomarsino**

Argentina

**Markus Laimer**

Switzerland

**Leslie Laing**

Canada

**Pietro Lampertico**

Italy

**Athene Lane**

UK

**Roger Langford**

UK

**Jost Langhorst**

Germany

**Li Xing Lao**

Hong Kong

**Sarah Larney**

Australia

**Marissa Lassere**

Australia

**Jorgen Lauridsen**

Denmark

**Jens Lauritsen**

Denmark

**Maurice Laville**

France

**Julia Lawton**

UK

**Jimmy Le**

USA

**M Le Guen**

France

**Joanna Leaviss**

UK

**Robert Lebeau**

USA

**Annie LeBlanc**

USA

**Myeong Soo Lee**

Korea, South

**Seung Eun Lee**

Korea, South

**Minji Lee**

USA

**Jun-Hwan Lee**

Korea, Republic of

**Chii-Ming Lee**

Taiwan

**Paul Lee-Archer**

Australia

**Jungtae Leem**

Korea, South

**Francois Lemaire**

France

**Gilles Lemesle**

France

**Dennis Lendrem**

UK

**Emmanuel Lesaffre**

Belgium

**Kate Leslie**

Australia

**Steff Lewis**

UK

**Xinyan Li**

China

**Min Li**

Hong Kong

**Ping Li**

China

**Yi-Heng Li**

Taiwan

**Shaojung Li**

Taiwan

**Fan-Rong Liang**

China

**Chien-Chang Liao**

Taiwan

**Richard Liebano**

Brazil

**Richard Lilford**

UK

**Zhixiu Lin**

Hong Kong

**Yang-Hua Lin**

Taiwan

**Augusto Litonjua**

USA

**Cun-Zhi Liu**

China

**Jian-Ping Liu**

China

**Zhihong Liu**

China

**Lizhou Liu**

New Zealand

**Teresa Liu-Ambrose**

Canada

**Christopher Lo**

Canada

**Dileep Lobo**

UK

**Carl Lombard**

South Africa

**Dustin Long**

USA

**Kirsty Loudon**

USA

**Chuanjian Lu**

China

**Elizabeth Lutge**

South Africa

**Jinhui Ma**

Canada

**Graeme MacLennan**

UK

**Julia Mader**

Austria

**Michael Maes**

Australia

**Lisa Maguire**

UK

**Dermot Maher**

Switzerland

**Govind Makharia**

India

**Khurram Malik**

USA

**Laxmaiah Manchikanti**

USA

**Laurent Mandelbrot**

France

**Ulrich Mansmann**

Germany

**Lamberto Manzoli**

Italy

**Louise Maranda**

USA

**Branka Marinovic**

Croatia

**Amelia Marques**

Brazil

**Zoe Marshman**

UK

**Seth Martin**

USA

**Marrissa Martyn-St James**

UK

**Mihaela Matei**

France

**Yoichi Matsui**

Japan

**Luciana Matsutani**

Brazil

**Richard Mattick**

Australia

**Gian Carlo Mattiucci**

Italy

**Nancy Mayo**

Canada

**Craig McBride**

Australia

**Corrigan McBride**

USA

**Catriona McDaid**

UK

**Patrick McEwan**

USA

**Aoife McGarvey**

Australia

**Mark McGovern**

USA

**Alison H McGregor**

UK

**Brian McGuire**

Ireland

**Amanda McIntyre**

Canada

**Christopher McKevitt**

UK

**Noreen Mdege**

UK

**Arianeb Mehrabi**

Germany

**Roberto Méndez-Sánchez**

Spain

**Sarah Meneses**

Brazil

**Alain Mercat**

France

**Hanna Merk**

Sweden

**Sally Merry**

New Zealand

**Luc Mertens**

Canada

**David Metcalfe**

UK

**William Meurer**

USA

**Pierre-Luc Michaud**

Canada

**Nicola Mills**

UK

**Yosuke Miyashita**

USA

**Somaia Mohamed**

USA

**Stephen Möhlhenrich**

Germany

**Ben Willem Mol**

Netherlands

**Helen Monaghan**

Australia

**Toni Monleon**

Spain

**Leonard Montenij**

Netherlands

**In Seok Moon**

Korea, South

**Sarah Moore**

Switzerland

**Iain Moppett**

UK

**Mariana Moreira da Silva**

Brazil

**Gemma Morgan**

UK

**William Mortenson**

Canada

**Othmar Moser**

Germany

**Loren Mowszowski**

Australia

**Thomas Mücke**

Germany

**Mark Mullee**

UK

**Keith Muller**

USA

**Praveen Mummaneni**

USA

**Gordon Murray**

UK

**David Musch**

USA

**Charles Nager**

USA

**Mor Nahum**

USA

**Madhavan Nair**

India

**Zoher Naja**

Lebanon

**Mohammad Javad Nasiri**

Iran

**Helene Korvenius Nedergaard**

Denmark

**Melissa Neuman**

UK

**Corine Awonja Ngufor**

UK

**Michael Nicholson**

UK

**Marc Nickels**

Australia

**Massimo Nicolo'**

Italy

**Remy Nizard**

France

**Devon Noonan**

USA

**Gill Norman**

UK

**Seth Norrholm**

USA

**Saad Nseir**

France

**Kevin OBrien**

UK

**Ayodele Odutayo**

Canada

**Akiko Okifuji**

USA

**Sjurdur Olsen**

Denmark

**Stefano Omboni**

Italy

**Robert O'Neill**

USA

**Albert Oriol**

Spain

**Anthony Ormerod**

UK

**Augustine Osman**

USA

**Katja Ott**

Germany

**Petronella Ottevanger**

Netherlands

**Nathan Leon Pace**

USA

**Salvatore Paiella**

Italy

**Montse Palacio**

Spain

**Rebecca Palmer**

UK

**Amanda Palmer**

USA

**Angeliki Papadaki**

UK

**Sophia Papadakis**

Canada

**Kosmas Paraskevas**

Greece

**Martyn Parker**

UK

**Christopher Parshuram**

Canada

**Nick Parsons**

UK

**Sujata Patil**

USA

**Oliver Peacock**

UK

**Elizabeth Pearce**

USA

**Dominic Pearson**

UK

**Claudia Pedroza**

USA

**Jose Penalvo**

USA

**Wenceslao Penate**

Spain

**Stephen Pereira**

UK

**Nuria Perez-Alvarez**

Spain

**Irene Pericot-Valverde**

Spain

**Oliver Perkin**

UK

**Julien Peron**

France

**Sean Perrin**

UK

**Elodie Perrodeau**

France

**Daniel Christopher Perry**

UK

**Florian Peters**

Germany

**Ron Peters**

Netherlands

**Andrew Peterson**

USA

**Phillip Peterson**

USA

**Mila Petrova**

UK

**Lira Pi**

USA

**Gilda Piaggio**

France

**Mirjana Pibernik-Okanovic**

Croatia

**Jay Piccirillo**

USA

**Gisele Pickering**

France

**Stefan Pilz**

Austria

**Romain Pirracchio**

USA

**Marie Pitkethly**

UK

**Robert Platt**

Canada

**Jan Poelaert**

Belgium

**Tamara Poljicanin**

Croatia

**Robert Pollard**

USA

**Gregory Pond**

Canada

**Caridad Pontes**

Spain

**Raphael Porcher**

France

**Colin Powell**

UK

**Melanie Prague**

USA

**Andrew Prayle**

UK

**Jennifer Preston**

UK

**Melanie Price**

Australia

**Lindsay Prior**

UK

**Pascal Probst**

Germany

**Riccardo Proietti**

Italy

**Audrey Prost**

UK

**John Prowle**

UK

**Kristi Pruiksma**

USA

**Livia Puljak**

Croatia

**Nithima Purepong**

Thailand

**Im Quah-Smith**

Australia

**Jean-Pierre Quenot**

France

**Terence Quinn**

UK

**Terry Quinn**

UK

**Virginia Quinn**

USA

**Praveer Rai**

India

**Nicole Rankin**

Australia

**Madhumathi Rao**

USA

**Tim Rapley**

UK

**Petra Rauchhaus**

UK

**David Ray**

UK

**Julie Redfern**

Australia

**Stefania Redolfi**

France

**George Reed**

USA

**Barnaby Reeves**

UK

**Jean Marc Regimbeau**

France

**Jean-Philippe Regnaux**

France

**Jean Reignier**

France

**Cecile Remuzat**

France

**Jean-Sebastien Renaud**

Canada

**Suzanne Richards**

UK

**Jo Rick**

UK

**Philip Riley**

UK

**Robert Rintoul**

UK

**Jamie Roberts**

USA

**Jamie Roberts**

USA

**Thompson Robinson**

UK

**Helen Rodgers**

UK

**Isabel Rodriguez-Barraquer**

USA

**Pierre Rompré**

Canada

**Nitin Roper**

USA

**Stephen Rosenstiel**

USA

**Joao Ruaro**

Brazil

**Julia Rucklidge**

New Zealand

**Belén Ruiz-Antorán**

Spain

**Jacqueline Rushby**

Australia

**Everardo Saad**

Brazil

**Christoph Saely**

Austria

**Saruta Saengtipbovorn**

Thailand

**Joaquín Sáez-Peñataro**

Spain

**Raphael Saginur**

Canada

**Christopher Saigal**

USA

**Daisuke Sakai**

Japan

**Brodie Sakakibara**

Canada

**Ian Saldanha**

USA

**Gil Salles**

Brazil

**Nir Salomon**

Israel

**Judith Samonigg**

Austria

**Arantxa Sancho-Lopez**

Spain

**Joseph Sanford**

USA

**Karla Santo**

Australia

**Sandra Saperstein**

USA

**Jan M Sargeant**

Canada

**David Saunders**

Thailand

**Benedicte Sautenet**

France

**Dirk Schaedler**

Germany

**Roberta Scherer**

USA

**Michael Schlussel**

UK

**Thomas Schmidt**

Germany

**Marguerite Schneider**

South Africa

**Andreas Schnitzbauer**

Germany

**Rob Scholten**

Netherlands

**Vanessa Scholtes**

Netherlands

**David Schriger**

USA

**Peter Schwarz**

Germany

**Falk Schwendicke**

Germany

**Philip Sedgwick**

UK

**Paul Seed**

UK

**Hanna Seidling**

Germany

**Patrick Sequeira-Byron**

Switzerland

**David Shade**

USA

**Haleema Shakur**

UK

**Hongcai Shang**

China

**Surya Kant Sharma**

India

**Virginia Sharpe**

USA

**Zeeshan Sheikh**

Canada

**Johannah Shergis**

Australia

**Jian-Rong Shi**

China

**Maurice Sillen**

Netherlands

**Iveta Simera**

UK

**Greg Simon**

USA

**Ian Sinha**

UK

**Trygve Skonnord**

Norway

**Susan Slaughter**

Canada

**Nicola Small**

UK

**Valerie Smith**

Ireland

**Scott Smith**

USA

**Claire Snowdon**

UK

**Ameenat Lola Solebo**

UK

**Daniel Solomon**

USA

**Harald Sourij**

Austria

**Manon Spaander**

Netherlands

**Anne Speckens**

Netherlands

**Dominique Sprumont**

Switzerland

**Vinit Srivastava**

India

**Philip Stahel**

UK

**Nigel Stallard**

UK

**Zeno Stanga**

Switzerland

**Alisa Stephens Shields**

USA

**Fiona Stewart**

UK

**Mette Jensen Stochkendahl**

Denmark

**Jennifer Straatman**

Netherlands

**Daniel Strech**

Germany

**Stacey Su**

USA

**Carolyn Summerbell**

UK

**Yan Sun**

China

**Wei-Zen Sun**

Taiwan

**Hulya Sungurtekin**

Turkey

**Christina Surawy**

UK

**Cynthia Swarnalatha**

Canada

**Stephen Swisher**

USA

**Matthew Robert Sydes**

UK

**Monica Taljaard**

Canada

**Patricia Tang**

Canada

**David Tappin**

UK

**Martin Tauschmann**

UK

**Adrian Taylor**

UK

**Inna Tchivileva**

USA

**Ekmel Tezel**

Turkey

**Hiran Thabrew**

New Zealand

**Jay Thakkar**

Australia

**George Thanassoulis**

Canada

**Achilleas Thoma**

Canada

**Lucy Thomas**

Australia

**Simon Francis Thomsen**

Denmark

**Margaret Thorogood**

UK

**Kevin Thorpe**

Canada

**Lene Thorsen**

Norway

**Svetlana Tikhonova**

Canada

**Nickolai Titov**

Australia

**Angela Todd**

Australia

**Susan Todd**

UK

**Merran Toerien**

UK

**Palle Toft**

Denmark

**George Tomlinson**

Canada

**David Torgerson**

UK

**Ferran Torres**

Spain

**Anna Towers**

Canada

**Viet-Thi Tran**

France

**Amy Traylor**

USA

**Shaun Treweek**

UK

**Lazaros Tsalikis**

Greece

**Lucy Turner**

Canada

**James Turner**

UK

**Chris Twelves**

USA

**Jos Twisk**

Netherlands

**Nikolaos Tzemos**

UK

**Jacob Udell**

Canada

**Gerard Urrutia**

Spain

**Antonis Valachis**

Sweden

**Johanna van der Bom**

Netherlands

**Rieke Van der Graaf**

Netherlands

**Martinus Hendrikus Gerardus Maria van der Pas**

Netherlands

**Hidde P van der Ploeg**

Netherlands

**Bart van der Worp**

Netherlands

**Jeroen van Meijgaard**

USA

**Talitha Verhoef**

UK

**Helena Verkooijen**

Netherlands

**Renee Verwey**

Netherlands

**Andrew Vickers**

USA

**Mauro Vigan Ograve**

Italy

**Anna Virgili**

Italy

**Benno Von Bormann**

Thailand

**Alexander Vonbank**

Austria

**Kalpana Vora**

India

**Francois Vrtovsnik**

France

**Helen Wakeford**

UK

**Jens Waldmann**

Germany

**Peter Wall**

UK

**Sherrie Wallington**

USA

**Shu Wang**

China

**Jaw-Yuan Wang**

Taiwan

**James Wason**

UK

**Heather Waterman**

UK

**Fiona Webster**

Canada

**Ruth Webster**

Australia

**Odette Wegwarth**

Germany

**Charles Weijer**

Canada

**Christel Weiss**

Germany

**Carolina Weller**

Australia

**Patrick Wen**

USA

**Zehuai Wen**

China

**Keith Wheatley**

UK

**Angela White**

Canada

**Peter White**

UK

**Will Whiteley**

UK

**Patricia Whitfield**

New Zealand

**Christian Wiedermann**

Italy

**Lucas Wiegand**

USA

**Phil Wiffen**

UK

**Sally Louise Williams**

Italy

**Don Willison**

Canada

**Paul M Wilson**

UK

**Anne Wilson**

UK

**Jennifer Wingham**

UK

**Zoe Winters**

UK

**Michelle Wise**

New Zealand

**Nicholas Wise**

USA

**Charles Wiysonge**

South Africa

**Barbara Wizner**

Poland

**Janice Wong**

USA

**Kerry Woolfall**

UK

**Bo Wu**

China

**Justin Wu**

Hong Kong

**Yen-Wen Wu**

Taiwan

**Qian Wu**

USA

**Rudolf P Wüthrich**

Switzerland

**Jielai Xia**

China

**Bin Xu**

China

**Kyohei Yamaji**

Japan

**Yong-Qing Yang**

China

**Jie Yang**

China

**Zifeng Yang**

China

**Mingxiao Yang**

China

**D Yates**

UK

**Amelie Yavchitz**

France

**Bridget Young**

UK

**Jing Yuan**

Hong Kong

**Nese Yuksel**

Canada

**Joanna Zakrzewska**

UK

**Ann Zauber**

USA

**William Zempsky**

USA

**Pamela Zengel**

Germany

**Yong Zhang**

China

**Qinhong Zhang**

China

**Zhigang Zhang**

China

**Ping Zhao**

China

**Ling Zhao**

China

**Cuihong Zheng**

China

**Linda LD Zhong**

Hong Kong

**Linda Zhong**

Hong Kong

**Fen Zhou**

China

**Bingmei Zhu**

China

**Feng-Cai Zhu**

China

**Jose Zubizarreta**

USA

**Massarat Zutshi**

USA

**Merrick Zwarenstein**

Canada

